# Obesity, lifestyle risk-factors, and health service outcomes among healthy middle-aged adults in Canada

**DOI:** 10.1186/1472-6963-12-238

**Published:** 2012-08-04

**Authors:** David A Alter, Harindra C Wijeysundera, Barry Franklin, Peter C Austin, Alice Chong, Paul I Oh, Jack V Tu, Therese A Stukel

**Affiliations:** 1 Institute for Clinical Evaluative Sciences, 2075 Bayview Avenue, Toronto, ON, Canada; 2 The Cardiac Rehabilitation and Secondary Prevention Program, Toronto Rehabilitation Institute, Toronto, Canada; 3 The Schulich Heart Centre and the Clinical Epidemiology Unit of Sunnybrook Health Science Centre, Toronto, Canada; 4 The Li Ka Shing Knowledge Institute of St. Michaels’ Hospital, Department of Medicine, Toronto, Canada; 5 Cardiac Rehabilitation and Exercise Laboratories, William Beaumont Hospital, Royal Oak, 3601 W. 13 Mile Rd, Royal Oak, MI, 48073, USA; 6 Department of Health Policy, Management and Evaluation, University of Toronto, Toronto, ON, Canada; 7 Toronto Rehabilitation Institute, 345 Rumsey Road, Toronto, ON, M4G 1R7, Canada; 8 William Beaumont Hospital, Royal Oak, 3601 W. 13 Mile Rd, Royal Oak, MI, 48073, USA

**Keywords:** Obesity, Risk-factors, Health service utilization, Health care expenditures, Cohort study, Outcomes

## Abstract

**Background:**

The extent to which uncomplicated obesity among an otherwise healthy middle-aged population is associated with higher longitudinal health-care expenditures remains unclear.

**Methods:**

To examine the incremental long-term health service expenditures and outcomes associated with uncomplicated obesity, 9398 participants of the 1994–1996 National Population Health Survey were linked to administrative data and followed longitudinally forward for 11.5 years to track health service utilization costs and death. Patients with pre-existing heart disease, those who were 65 years of age and older, and those with self-reported body mass indexes of <18.5 kg/m^2^ at inception were excluded. Propensity-matching was used to compare obesity (+/− other baseline risk-factors and lifestyle behaviours) with normal-weight healthy controls. Cost-analyses were conducted from the perspective of Ontario’s publicly-funded health care system.

**Results:**

Obesity as an isolated risk-factor was not associated with significantly higher health-care costs as compared with normal weight matched controls (Canadian $8,294.67 vs. Canadian $7,323.59, P = 0.27). However, obesity in combination with other lifestyle factors was associated with significantly higher cumulative expenditures as compared with normal-weight healthy matched controls (CAD$14,186.81 for those with obesity + 3 additional risk-factors vs. CAD$7,029.87 for those with normal BMI and no other risk-factors, P < 0.001). The likelihood that obese individuals developed future diabetes and hypertension also rose markedly when other lifestyle factors, such as smoking, physical inactivity and/or psychosocial distress were present at baseline.

**Conclusions:**

The incremental health-care costs associated with obesity was modest in isolation, but increased significantly when combined with other lifestyle risk-factors. Such findings have relevance to the selection, prioritization, and cost-effective targeting of therapeutic lifestyle interventions.

## Background

Obesity accounts for nearly $80 billion per year and between 2% and 3% of total health care expenditures in North America [1] a 6% to 45% relative increase in health care expenditures when compared with age and gender matched normal weight populations [2]. The incremental increases in population-attributable costs have been ascribed predominantly to the development of long-term complications and their associated impacts on hospital-, physician service-, and medication-related expenditures [3-6]. Recent studies suggest that the growth in health system expenditures attributable to obesity will, in fact, surpass cigarette smoking, and become the predominant public health issue in North America [7]

Nonetheless, there are several limitations associated with previous research. First, few studies have disentangled the independent effects of obesity from other risk-factors and disease-related complications that arise over time [1]. Such studies require longitudinal follow-up of a population who are free of cardiovascular disease at inception, so that forthcoming health system expenditures reflect the emergence of new incident diseases that occur during follow-up rather than pre-existing diseases already present at baseline. Second, obesity’s associations with health outcomes may vary according to age. For example, obesity may be more strongly associated with diabetes, hypertension, and mortality among middle-aged adults than among the elderly [8-12]. Third, the impact of obesity on health-care expenditures may vary according to the presence or absence of other concomitant lifestyle behaviours. For example, the cost-implications associated with obesity may be significantly greater among a physically inactive individual than an individual who is physically active [13-16]. In order to delineate costs attributable to obesity itself, analyses must match or adjust for other lifestyle factors. Finally, the feasibility, effectiveness, and cost-effectiveness of obesity-related public health policy interventions may be undermined by the tremendous burden of obesity in the population and by the heterogeneity in risk-factors profiles within obese populations [17-20]. Delineating high-risk, high-cost lifestyle risk-factor combinations may help direct, focus, and target policy lifestyle interventions for obesity.

Accordingly, the objective of our study was to examine the cumulative longitudinal health system expenditures and outcomes associated with obesity among a cohort of middle-aged adults in Ontario Canada, all of whom were free from cardiovascular disease at inception, and to compare these data with propensity-matched normal weight healthy controls. Our analyses were conducted from the perspective of Ontario’s publically funded health care system, which covers the costs of medical health care delivery for all of it’s residents, as well as the costs for medications among individuals’ 65 years of age and older.

## Methods

### Data sources

The Ontario Health Survey (OHS) was part of the 1996/1997 National Population Health Survey (NPHS) [21] involving all Canadian provinces. OHS participants were identified by telephone through random digit dialing. One adult from each contacted household was asked to give information about health care utilization, chronic health problems, social characteristics, and individual and cumulative income levels for all inhabitants. The survey excluded persons living in Indian Reserves, Canadian Forces bases, some remote areas of Ontario, and in institutions or collective dwellings. Homeless persons and those without access to a telephone were also excluded. Linkage of the initial OHS sample to administrative databases required patients consent to link their health card numbers to a population file in order to track downstream health service utilization and mortality. While 90% of the initial survey sample provided consent to link, valid health card numbers were present in only 62% of the initial survey sample. When compared to those in whom data linkage was not feasible, the linked sample was older, more affluent, and had more co-existing illnesses. However, the absolute differences between linked and unlinked samples were small, and have been reported elsewhere [22]. Physician visits were tracked through the Ontario physician’s claims databases (Ontario Health Insurance Plan), while cardiac procedures and hospitalizations were obtained through hospital discharge records (Canadian Institutes of Health Information), and mortality was identified through a vital statistics registry (Registered Persons Database). Additional details regarding the linked population-based sample have been reported elsewhere [22]. Costs related to drug claims were available through the Ontario Drug Benefits (ODB) formulary once individuals became ODB eligible (i.e., reaching the age of 65 years during follow-up or were in special programs such as welfare). The subsequent development of diabetes and hypertension were obtained using the validated Ontario Diabetes and Ontario Hypertension Databases [23,24].

### Study population

**Figure 1:**
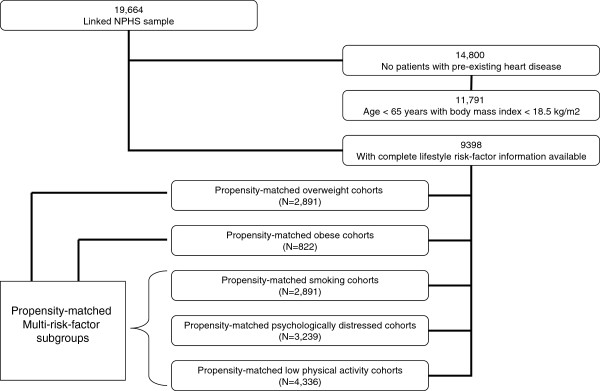
Study sample eligibility subdivided according to subgroups used in propensity analyses NPHS = National Population Health Survey.

To ensure we examined a relatively homogenous disease-free middle-aged population, we excluded all individuals with known heart disease at baseline (as identified using both self-report and retrospectively linked administrative health records extending historically to 1988), those ≥ 65 years of age, and underweight individuals (self-reported body mass indexes [BMI] < 18.5 kg/m^2^) (Figure 1), given that the development of chronic disease and health care expenditures in these subgroups are likely mediated by factors other than obesity alone [8-11].

### Baseline socioeconomic, ethnicity, demographic factors

Survey respondents reported on total annual household income as an 11-level categorical variable, which was re-categorized into three income-subgroups as has been done previously [22,25]. These include: low income (< $30,000 per year); intermediate income ($30,000-$49,999 per year); and high income (≥ $50,000 per year). Information on ethnicity was obtained through self-report from one or more categories of 12 ethnic/racial subgroups as defined by the Canadian Census [26]. For our purposes, ethnic/racial data were re-aggregated a priori into a binary predictor variable of Caucasian or non-Caucasian.

### Baseline clinical risk

Prior hospitalizations and baseline comorbidity were assessed using both self-report and retrospectively linked administrative databases extending historically to 1988 (i.e., look-back period of 8 years per patient prior to cohort inception). For the purposes of our study, high alcohol consumption, defined as consuming ≥ 2 drinks per day, served as a surrogate clinical marker rather than as a lifestyle risk-factor (see below) given its association with other chronic diseases, including an increased risk of cancer in women [27]. However, re-classifying alcohol consumption as a lifestyle rather than a clinical factor did not alter our results.

### Obesity and overweight

Individuals were classified as obese or overweight based upon BMI as derived using self-reported height and weight. Obesity was defined as a BMI ≥ 30 kg/m^2^; overweight was defined as a BMI of 25.0-29.9 kg/m^2^. Normal BMI was defined as derived measures between 18.5-24.9 kg/m^2^.

### Other lifestyle risk-factors

Self-reported physical activity (defined as the number of days per month exercising for ≥ 15 minutes per session), smoking status, and psychosocial distress served as other lifestyle risk-factor comparators for our study. For the purposes of this study, a sedentary lifestyle was defined as a frequency of physical activity that fell below the median for the study sample (i.e., < 17 days per month). Psychosocial distress was evaluated using the OHS derived distress scale, which is based on a subset of items from the Composite International Diagnostic Interview in which scores ranged from 0 to 24, with higher scores indicating greater degrees of distress. [26] Psychosocial distress was defined as scores above the sample median (> 2) [28]. Varying threshold values to define sedentary lifestyle, psychosocial distress, or both, did not meaningfully alter our results.

### Primary outcomes: health care utilization expenditures

Cumulative health care expenditures served as the primary outcome. In accordance with the Canadian Agency for Drugs and Technologies in Health which recommends that Canadian cost-analysis and/or comparative effectiveness studies are assessed from the perspective of the publically funded health care system [29], we focused our Ontario cost-analyses to medically insured services. We utilized a bottom-up approach when tallying the health service costs for individuals over the entire study duration of 11.5 years. Health service costs consisted of all primary and specialty-care physician visits, emergency-room visits, hospitalizations, invasive cardiac testing, revascularization procedures, and medication costs once individuals reached the age of 65 years (or if incomes fell below the poverty thresholds) during follow-up. Medication costs for individuals younger than 65 years old where not examined given that such expenditures are not covered by Ontario’s publicly funded health care system. Costs related to hospitalizations and emergency room visits were estimated using the resource intensity weight (RIW) methodology, whereby the cost is the product of the RIW associated with a particular case and the average cost per weighted case for Ontario [30]. The Ontario Case Costing Initiative was used to estimate the costs related to percutaneous coronary intervention and coronary artery bypass surgery [31]. All costs were adjusted to 2006 Canadian dollars, using the Bank of Canada Consumer Price Index.

### Secondary outcomes

Secondary outcomes included all-cause mortality, the occurrence of acute myocardial infarction or death, and the new development (incident cases) of diabetes and/or hypertension during the follow-up period as identified using the validated Ontario Diabetes and Ontario Hypertension Databases [23,24].

### Analytic techniques

A series of propensity-score-matched samples were constructed in which cumulative costs associated subjects with obesity were compared against cumulative costs associated with normal weight matched controls. Propensity analyses allows for the balancing of baseline characteristics between exposed (e.g.., obese) and un-exposed (e.g., normal weight controls) individuals. The advantage of propensity analyses is that outcome differences can be more easily causally attributed to exposure rather than to confounding factors since the latter variables were balanced between the two groups in a manner that is analogous to that of a randomized clinical trial. In this study, each exposed individual (e.g., an individual with obesity) was matched in 1:1 fashion to a healthy normal weight control, based on a series of important confounders such as age, gender, socioeconomic status, smoking, physical activity, psychosocial stress, and comordidity. The difference in health care costs between the exposed and unexposed “matched pair” is then determined. The differences in costs between each matched pair were then tallied and averaged throughout the entire matched-cohort. Costs associated with other risk-factors were also compared against costs associated with their propensity-matched non-risk factor counterparts (e.g.,. smokers were compared with non-smoker match controls). When combining multiple baseline risk-factors together, healthy subjects with no risk-factors present at baseline served as propensity-matched controls (Figure 1). Each person’s propensity score was based on the following variables: age, gender, ethnicity, socioeconomic status, prior hospitalizations, pre-existing diabetes, pre-existing hypertension, pre-existing depression, alcohol consumption, physical activity level (where appropriate), psychosocial distress (where appropriate), and smoking (where appropriate). Propensity-score matched samples were constructed by using greedy nearest-neighbour matching with calipers of width equal to 0.2 standard deviation of the logit of the propensity score [32,33]. Statistical significance was defined as two-tailed P < 0.05. Comparison of outcomes between each risk factor group and normal controls were made using paired *t*-Test and McNemar’s for continuous and dichotomous variables respectively [34]. A series of sensitivity analyses were conducted and are described in the results.

All analyses utilized SAS v9.2 statistical software (SAS Institute Inc Cary, NC, USA).

## Results

### Baseline data

Table 1 summarizes selected characteristics of patients who were classified as obese, overweight, and normal weight at baseline. The obese cohort was significantly older and had a significantly higher prevalence of diabetes and hypertension; however, the prevalence of these risk factors was low (2.5% and 2.9%, respectively). Most subjects had at least one additional lifestyle risk-factor beyond increased BMI, with sedentary lifestyle being the most common, which had a significantly greater prevalence among obese than non-obese subgroups (P < 0.001). Over 30% of patients had multiple risk-factors (≥ 2) independent of BMI.

**Table 1:** **Baseline characteristics before propensity matching comparing individuals who were obese (BMI > 30 kg/m**^**2**^**) with normal weight individuals (BMI 18.5-24.9 kg/m**^**2**^**), and comparing individuals who were overweight (BMI 25–29.9kg/m**^**2**^**) with normal weight persons (BMI 18.5-24.9 kg/m**^**2**^**)**

		**Obese (N = 1,363)**	**Overweight (N = 3,375)**	**Normal (N = 4,660)**	P value^*^
Socio-ethno-demographic	Male (%)	718 (52.7)	2,136 (63.3)	1,858 (39.9)	<0.001
	Mean age (SD)	42.56	41.83	37.9 (11.5)	<0.001
		(11.56)	(11.69)		
	Caucasian (%)	1296 (95.1)	3154 (93.5)	4247 (91.1)	<0.001
	High income (%)	553 (40.6)	1581 (46.8)	2024 (43.4)	0.72
	Intermediate income (%)	398 (29.2)	979 (29.0)	1309 (28.1)	0.32
	Low income (%)	412 (30.2)	815 (24.2)	1327 (28.5)	0.54
Pre-existing disease	Prior hospitalizations (%)	502 (38.8)	1033 (30.6)	1670 (35.8)	0.19
	Diabetes (%)	34 (2.5)	62 (1.8)	41 (0.88)	<0.001
	Hypertension (%)	39 (2.9)	42 (1.2)	25 (0.54)	<0.001
	Prior depression (%)	25 (1.8)	32 (0.95)	63 (1.35)	0.60
	High alcohol consumption^†^ (%)	323 (23.7)	1070 (31.7)	1226 (26.3)	0.61
Risk-factors	Current smoking (%)	384 (28.2)	998 (29.6)	1653 (35.5)	<0.001
	Sedentary^‡^	777 (57.0)	1743 (51.6)	2277 (48.9)	<0.001
	Psychological distress^§^	518 (38.0)	1098 (32.5)	1713 (36.8)	0.42
0 other risk-factors	None	302 (22.2)	837 (24.8)	1104 (23.7)	0.002
1 other risk-factor	Sedentary-only^‡^	332 (24.4)	831 (24.6)	897 (19.3)	
	Distressed-only^§^	131 (9.6)	359 (10.6)	526 (11.3)	
	Smoking-only	79 (5.8)	269 (8.0)	438 (9.4)	
2 other risk-factors	Sedentary + distressed	214 (15.7)	350 (10.4)	480 (10.3)	
	Sedentary + smoking	132 (9.7)	340 (10.1)	508 (10.9)	
	Distressed + smoking	74 (5.4)	167 (5.0)	315 (6.8)	
3 other risk-factors	Sedentary + distressed + smoking	99 (7.3)	222 (6.6)	392 (8.4)	

^*^ Mantel-Haenszel Chi-Square for dichotomous outcomes; least squares regression with 1 degree of freedom of continuous outcomes (e.g., age).

^†^ High alcohol consumption is defined as consumption exceeding the median level for the entire cohort (regardless of subgroup and prior to propensity matching) and consists of consuming ≥ 2 drinks per day.

^‡^ Sedentary lifestyle was defined as a frequency of physical activity that fell below the median for the study sample (i.e.,. < 17 days per month of exercising for ≥ 15 minutes per session).

^§^ High psychological distress is defined as the number of individuals whose distress scores, as measured using the Ontario Health Survey derived distress scale exceeding the median for the entire cohort (a score of >2) regardless of subgroup and prior to propensity matching).

### Relationship between obesity and long-term expenditures

The median health care expenditures experienced by an individual throughout the study period was [CAD] $3182.54 (IQR = $8881.81; Q25% = $1023.15 and Q75% = $9904.96]. Table 2 shows the baseline characteristics of obese (and overweight) populations as compared with normal weight controls, following propensity-matching where the distribution of baseline covariates were well-balanced between the two groups. The baseline characteristics associated with smokers, distressed, and sedentary populations are shown in Additional file 1. Although there was a trend toward higher health care expenditures, obesity was not associated with significantly higher cumulative costs as compared with propensity-matched normal weight controls (CAD$8,294.67 vs. CAD$7,323.59 per person over 11.5 years of follow-up, P = 0.27). In contrast, those categorized as smokers or psychologically distressed had significantly higher health care expenditures over 11.5 years of follow-up than their corresponding non-smoking, non-distressed propensity-matched controls (Table 3).

**Table 2:** **Baseline characteristics after propensity matching comparing individuals who were obese (BMI > 30 kg/m**^**2**^**) with normal weight individuals (BMI 18.5-24.9 kg/m**^**2**^**), and comparing individuals who were overweight (BMI 25–29.9kg/m**^**2**^**) with normal weight persons (BMI 18.5-24.9 kg/m**^**2**^**)**

	**Overweight (N = 2,891)**	**Normal weight (N = 2,891)**	**Standardize differences of the mean**	**Obese (N = 822)**	**Normal weight (N = 822)**	**Standardized difference of the mean**
Male (%)	1,439 (49.7)	1,429 (49.3)	0.01	340 (41.4)	346 (42.1)	0.01
Mean age (SD)	39.2 (11.7)	39.2 (11.6)	0.00	39.4 (11.5)	38.6 (11.4)	0.05
Caucasian (%)	2,681 (92.6)	2,699 (93.2)	0.02	748 (91.0)	757 (92.1)	0.04
High income (%)	1,299 (44.9)	1,277 (44.1)	0.01	357 (43.4)	378 (46.0)	0.05
Intermediate income (%)	845 (29.2)	854 (29.5)	0.00	229 (27.9)	231 (28.1)	0.01
Low income (%)	752 (26.0)	765 (26.4)	0.02	236 (28.7)	236 (28.7)	0.06
Prior hospitalizations (%)	945 (32.7)	907 (31.4)	0.03	290 (35.3)	266 (32.4)	0.06
High alcohol consumption^*^ (%)	842 (29.1)	823 (28.4)	0.01	209 (25.4)	219 (25.4)	0.03
Diabetes (%)	35 (1.2)	26 (0.9)	0.03	10 (1.2)	11 (1.3)	0.01
Hypertension (%)	23 (0.8)	13 (0.4)	0.04	8 (1.0)	2 (0.2)	0.09
High depression (%)	32 (1.1)	33 (1.1)	0.00	12 (1.5)	15 (1.8)	0.03
Current smoking (%)	963 (33.3)	933 (32.2)	0.02	260 (31.6)	220 (26.8)	0.11
Sedentary^†^	1,478 (51.1)	1,475 (51.1)	0.00	414 (50.4)	405 (49.3)	0.02
Psychological distress^‡^	1,009 (34.8)	993 (34.3)	0.01	317 (38.6)	288 (35.0)	0.07

^*^ High alcohol consumption is defined as consumption exceeding the median level for the entire cohort (regardless of subgroup and prior to propensity matching) and consists of consuming ≥ 2 drinks per day.

^†^ Sedentary lifestyle was defined as a frequency of physical activity that fell below the median for the study sample (i.e.,. < 17 days per month of exercising for ≥ 15 minutes per session).

^‡^ High psychological distress is defined as the number of individuals whose distress scores, as measured using the Ontario Health Survey derived distress scale exceeding the median for the entire cohort (a score of >2) regardless of subgroup and prior to propensity matching).

**Table 3:** Health care expenditures among obese, overweight, smokers, distressed, and those with sedentary lifestyles (each compared to their corresponding propensity-matched controls)^*^

**Health service expenditures CAD $**	**Obese (BMI ≥ 30.0 kg/m**^**2**^**) (N = 812)**	**Matched normal weight controls (BMI: 18.5-24.9 kg/m**^**2**^**)(N = 812)**	**P value**
Total cost (SD)	8,294.67 (19,836.89)	7,323.59 (16,319.45)	0.27
Hospitalization costs (SD)	5,579.27 (17,703.04)	4,817.31 (13,976.07)	0.33
Physician visit costs (SD)	1,347.11 (1,503.19)	1,298.95 (1,374.94)	0.49
Drug costs (SD)	4,166.72 (5,501.10)	4,635.79 (6,421.78)	0.35
Cardiac procedural costs (SD)	304.25 (2,017.39)	157.05 (1,401.07)	0.08
	**Overweight (BMI: 25–29.9 kg/m**^**2**^**)(N = 2,896)**	**Matched normal weight controls (BMI: 18.5-24.9 kg/m**^**2**^**)(N = 2,896)**	**P value**
			
Total cost (SD)	7,138.16 (18,443.25)	6,866.16 (17,697.33)	0.56
Hospitalization costs (SD)	4,678.35 (16,460.79)	4,362.34 (15,810.42)	0.46
Physician visit costs (SD)	1,186.89 (1,107.21)	1,226.22 (1,328.81)	0.21
Drug costs (SD)	4,408.64 (6,087.27)	4,863.85 (6,327.79)	0.12
Cardiac procedural costs (SD)	199.94 (1,767.63)	188.67 (1,602.24)	0.80
	**Smokers** (N = 2,966)	**Matched non-smoker controls** (N = 2,966)	
Total cost (SD)	8,626.50 (21,597.33)	7,299.83 (19,696.90)	0.004
Hospitalization costs (SD)	6,001.27 (19,183.45)	4,779.86 (17,320.47)	0.01
Physician visit costs (SD)	1,269.20 (1,365.92)	1,242.50 (1,279.84)	0.42
Drug costs (SD)	5,310.55 (6,945.39)	4,841.21 (6,643.73)	0.33
Cardiac procedural costs (SD)	334.81 (2,216.06)	164.71 (1,501.69)	<0.001
	**Distressed** (N = 3,239)	**Matched non-distressed controls** (N = 3,239)	
Total cost (SD)	8,454.12 (20,932.08)	7,131.86 (19,073.29)	0.007
Hospitalization costs (SD)	5,575.40 (18,489.80)	4,726.94 (17,050.19)	0.05
Physician visit costs (SD)	1,453.16 (1,552.62)	1,168.33 (1,125.90)	<0.001
Drug costs (SD)	5,547.66 (7,210.97)	4,931.83 (6,920.64)	0.37
Cardiac procedural costs (SD)	249.56 (1,898.72)	192.45 (1,654.15)	0.20
	**Sedentary** (N = 4,302)	**Matched non-sedintary controls**(N = 4,302)	
Total cost (SD)	8,087.56 (19,681.31)	7,846.43 (20,749.90)	0.58
Hospitalization costs (SD)	5,224.70 (17,026.70)	5,111.80 (18,627.41)	0.76
Physician visit costs (SD)	1,275.04 (1,248.92)	1,266.49 (1,309.95)	0.75
Drug costs (SD)	5,341.78 (6,878.87)	4,837.28 (6,565.49)	0.22
Cardiac procedural costs (SD)	294.54 (2,184.06)	194.21 (1,651.09)	0.22

^*^ High alcohol consumption is defined as consumption exceeding the median level for the entire cohort (regardless of subgroup and prior to propensity matching) and consists of consuming ≥ 2 drinks per day.

Sedentary lifestyle was defined as a frequency of physical activity that fell below the median for the study sample (i.e.,. < 17 days per month of exercising for ≥ 15 minutes per session).

Additional file2 illustrates the baseline characteristics after propensity matching for patients with multiple risk-factors. In contrast to isolated risk-factors, health-care expenditures were significantly greater among overweight and obese individuals as compared with normal weight healthy controls when multiple lifestyle risk-factors were present. For example, when combined with smoking and/or psychological distress, an overweight individual had costs that were approximately CAD$2000 greater over 11.5 years than did a matched normal weight individual with no other risk-factors present at baseline. Similarly, an obese individual with other lifestyle risk-factors present had between CAD$2,700 to CAD$7,000 higher costs over 11.5 years than did a matched normal weight individual with no other risk-factors present at baseline (Table 4).

**Table 4:** Outcomes among individuals who were overweight or obese and had other lifestyle risk-factors (as compared with propensity-matched controls)^*^

**Overweight (BMI 25.0-29.9 kg/m**^**2**^**)**	**Cumulative average costs (SD)**	**STD of the mean**^**†**^	**P value**	**Obese (BMI ≥ 30 kg/m**^**2**^**)**	**Cumulative average costs (SD)**	**STD of the mean**^**†**^	**P value**
***One lifestyle risk-factor***				***One lifestyle risk-factor***			
Overweight (N = 2,896)	7,138.16 (18,443.25)	0.02	0.56	Obese (N = 822)	8,294.67 (19,836.89)	0.05	0.27
Normal weight healthy matched controls (N = 2,896)	6,866.16 (17,697.33)			Normal weight healthy matched controls (N = 822)	7,323.59 (16,319.45)		
***Two lifestyle risk-factors***				***Two lifestyle risk-factors***			
Overweight + smoking (N = 971)	8,880.13 (19,637.38)	0.11	0.02	Obese + smoking (N = 381)	11,910.27 (25,381.14)	0.16	0.03
Normal weight healthy matched controls (N = 971)	6,826.64 (18,575.46)			Normal weight healthy matched controls (N = 381)	8,128.23 (21,826.60)		
Overweight + sedentary (N = 1418)	8,013.25 (16,471.07)	0.06	0.12	Obese + sedentary (N = 765)	12,329.83 (28,969.98)	0.15	0.003
Normal weight healthy matched controls (N = 1418)	6,917.95 (19,682.70)			Normal weight healthy matched controls (N = 765)	8,250.36 (24,459.08)		
Overweight + distressed (N = 1089)	8,594.73 (18,768.77)	0.11	0.007	Obese + distressed (N = 508)	12,788.78 (26,685.21)	0.22	<0.001
Normal weight healthy matched controls (N = 1089)	6,579.22 (16,900.57)			Normal weight healthy matched controls (N = 508)	7,546.57 (19,592.65)		
***Three lifestyle risk-factors***				***Three lifestyle risk-factors***			
Overweight + smoking + sedentary (N = 535)	9,216.40 (19,452.87)	0.10	0.08	Obese + smoking + sedentary (N = 229)	11,317.21 (22,818.74)	0.11	0.22
Normal weight healthy matched controls (N = 535)	7,190.30 (19,885.19)			Normal weight healthy matched controls (N = 229)	8,684.30 (24,431.00)		
Overweight + smoking + distressed (N = 380)	9,465.67 (19,412.22)	0.19	0.009	Obese + smoking + distressed (N = 167)	11,749.28 (23,476.53)	0.26	0.02
Normal weight healthy matched controls (N = 380)	6,026.03 (17,524.67)			Normal weight healthy matched controls (N = 167)	6,402.59 (17,631.10)		
Overweight + distressed + sedentary (N = 566)	8,393.17 (17,643.75)	0.12	0.04	Obese + distressed + sedentary (N = 302)	14,186.81 (30,536.44)	0.30	<0.001
Normal weight healthy matched controls (N = 566)	6,524.60 (13,856.13)			Normal weight healthy matched controls (N = 302)	7,029.87 (14,931.77)		

^*^ High alcohol consumption is defined as consumption exceeding the median level for the entire cohort (regardless of subgroup and prior to propensity matching) and consists of consuming ≥ 2 drinks per day. Sedentary lifestyle was defined as a frequency of physical activity that fell below the median for the study sample (i.e.,. < 17 days per month of exercising for ≥ 15 minutes per session). High psychological distress is defined as the number of individuals whose distress scores, as measured using the Ontario Health Survey derived distress scale exceeding the median for the entire cohort (a score of >2) regardless of subgroup and prior to propensity matching).

^†^STD of the mean = Standardized Difference of the Mean.

As sensitivity analyses, we examined the relationship between BMI and longitudinal health care expenditures using more traditional analytic techniques. We did so among the original sample of 11,791 individuals regardless of missing data, and among the subgroup of 9,398 individuals for whom complete information were available for all. Our results did not change. Using Ordinary Least Squares regression, age and female sex were the strongest predictors of costs, accounting for 5% of the variation in individual long-term expenditures (R2 = 0.005). BMI, when examined either crudely or after adjustment for age, gender, prior hospitalizations, and risk-factors, was not significantly associated with individual long-term health care expenditures (ß = 32.8, p = 0.49). A series of re-analyses in which we explored various cut-points of BMI (i.e.., BMI exceeding 25 kg/m2 or 30 kg/m2) and other analytic techniques including Poisson and Logistic regression (i.e.., with the latter having the binary outcome of higher vs. lower than median expenditures) yielded similar findings as above.

### Secondary clinical outcomes

Table 5 illustrates the relationship between obesity and secondary outcomes. Obese individuals had significantly higher rates of death (4.6% vs. 2.1% for obese vs. normal weight, P = 0.004) and AMI or death (6.1% vs. 2.8% for obese vs. normal weight, P = 0.001) as compared with normal weight propensity-matched controls. The development of incident diabetes and hypertension during follow-up were not significantly higher among obese individuals as compared with normal weight controls. However, when combined with at least one other lifestyle risk factor at baseline, obese individuals had a significantly higher rate of future (incident) diabetes and hypertension as compared with normal weight controls. For example, subjects who were obese, smokers, and sedentary had a nearly 9-fold higher rate of developing diabetes, a 2-fold higher rate of developing hypertension, and a 2-fold higher risk of experiencing an adverse outcome over the 11.5 year follow-up than did normal-weight non-smoker, non-sedentary matched control.

**Table 5:** The development of future (incident) diabetes, future (incident) hypertension, and death over 11.5 years of follow-up among obese individuals +/− other lifestyle risk-factors (as compared with propensity-matched healthy controls)^*^

**Risk-factors**	**Diabetes**	**STD of the mean**^**†**^	**P value**	**Hypertension**	**STD of the mean**	**P value**	**AMI or death**^‡^	**STD of the mean**	**P value**	**Death**	**STD of the mean**	**P value**
***One lifestyle risk-factor***												
Obese (N = 822)	7.2%	0.03	0.55	17.0%	0.07	0.16	6.1%	0.16	0.001	4.6%	0.14	0.004
Normal weight healthy matched controls (N = 822)	6.4%			14.6%			2.8%			2.1%		
***Two lifestyle risk-factors***												
Obese + smoking (N = 381)	21.3%	0.57	<0.001	26.8%	0.32	<0.001	8.9%	0.16	0.03	6.0%	0.08	0.25
Normal weight healthy matched controls (N = 381)	3.1%			14.2%			5%			4.2%		
Obese + sedentary (N = 765)	21.7%	0.49	<0.001	32.4%	0.39	<0.001	7.5%	0.09	0.07	5.4%	0.06	0.25
Normal weight healthy matched controls (N = 765)	5.4%			17.6%			5.4%			4.2%		
Obese + distressed (N = 508)	21.3%	0.55	<0.001	30.3%	0.33	<0.001	9.3%	0.13	0.04	7.3%	0.09	0.15
Normal weight healthy matched controls (N = 508)	3.7%			16.5%			5.9%			5.1%		
***Three lifestyle risk-factors***												
Obese + smoking + sedentary (N = 229)	23.6%	0.65	<0.001	28.8%	0.37	<0.001	10%	0.24	0.006	6.6%	0.14	0.13
Normal weight healthy matched controls (N = 229)	2.6%			14%			3.9%			3.5%		
Obese + smoking + distressed (N = 167)	21.6%	0.62	<0.001	25.1%	0.40	<0.001	9.6%	0.16	0.13	7.8%	0.12	0.22
Normal weight healthy matched controls (N = 167)	2.4%			10.2%			5.4%			4.8%		
Obese + distressed + sedentary (N = 302)	22.2%	0.56	<0.001	33.8%	0.33	<0.001	9.6%	0.16	0.01	7.3%	0.18	0.03
Normal weight healthy matched controls (N = 302)	4.0%			19.5%			5.0%			3.6%		

^*^High alcohol consumption is defined as consumption exceeding the median level for the entire cohort (regardless of subgroup and prior to propensity matching) and consists of consuming ≥ 2 drinks per day. Sedentary lifestyle was defined as a frequency of physical activity that fell below the median for the study sample (i.e.,. < 17 days per month of exercising for ≥ 15 minutes per session). High psychological distress is defined as the number of individuals whose distress scores, as measured using the Ontario Health Survey derived distress scale exceeding the median for the entire cohort (a score of >2) regardless of subgroup and prior to propensity matching).

^†^STD of the mean = Standardized Difference of the Mean.

^‡^AMI or death = Acute myocardial infarction or death.

## Discussion

Our findings suggest that obesity as an isolated risk-factor in a middle-aged population was not associated with significantly higher incremental health care expenditures. However, obesity when combined with other baseline lifestyle risk-factors (e.g., psychological distress, physical inactivity, and/or smoking) was associated with significant increased cumulative costs as compared with normal weight propensity-matched healthy controls (with no other lifestyle risk-factors present at baseline). The risk of developing diabetes and hypertension during follow-up increased with multiple lifestyle factors, and was associated with higher expenditures.

Until now, few studies have attempted to examine longitudinal costs associated with obese adult populations from a point in time which preceded cardiovascular disease and its related complications [1,35]. As a result, previous studies may have spuriously attributed costs of other risk-factors, other lifestyle behaviours and/or other chronic disease to obesity thereby overestimating obesity-related expenditures [36]. Such methodological limitations may explain why obesity has been associated with higher adverse events in some studies, and lower adverse events in others [9,37-40]. Differences in obesity-related outcomes (and their associated expenditures) are likely more dependent on the constellation of lifestyle risk-factor behaviours and their longitudinal progression to disease, than on excess adiposity, body weight, or body-mass index as identified at any single point in time.

Our methodological longitudinal cohort design which excluded patients with cardiovascular disease, older adults (≥ 65 years of age), and underweight individuals at inception, allowed for obesity-related expenditures to be compared against normal weight controls from an inception point in which most individuals had yet developed disease-related complications. Moreover, the implementation of a propensity-matched design further balanced baseline characteristics, thereby allowing obesity to be examined within the context of other lifestyle factors thereby minimizing confounding. Such methods may have explained why obesity, when examined as an isolated risk-factor in a middle-aged population, failed to be associated with significant incremental expenditures as compared with matched controls.

Our results also demonstrated that incremental cumulative health care expenditures associated with body habitus rose substantially when BMI ≥ 25.0 kg/m^2^ was combined with other lifestyle risk-factors. For example, overweight and obese individuals who had 3 other lifestyle risk-factors (i.e.., sedentary lifestyle, cigarette smoking, psychologically distressed) had on average, $1,868 and $7,156 higher total expenditures respectively over 11.5 years of follow-up, than did propensity-matched normal weight healthy controls. In this respect, our results are consistent with others demonstrating the incremental cost implications associated with multiple as compared with isolated risk-factors [41].

Despite having only modest economic consequences, our results reaffirmed the importance of obesity as an isolated prognostic risk-factor of clinical outcomes of death, acute myocardial infarction, diabetes, and hypertension [42]. However, the development of chronic diseases (i.e.., incident diabetes and hypertension) as with the risk of death or acute myocardial infarction rose markedly when individuals with obesity had other adverse lifestyle factors already present at baseline. As with costs above, the longitudinal clinical risks associated obesity depended upon other pre-existing lifestyle factors - - a finding consistent with other studies [43-46].

We believe our study highlights the merits of risk -screening and risk-stratification using global health risk assessment tools that capture multiple lifestyle behaviours. For example, in our study, obese populations could be further stratified into higher risk, higher cost subgroups, through the identification of other adverse lifestyle behaviours. The broader implementation of health risk-assessments, which can identify and stratify individuals based on several lifestyle risk-factors may theoretically allow for more effective and cost-effective targeting of obese populations for therapeutic lifestyle interventions. Based on our study results, such targeted populations should include those individuals who are also physically inactive, who smoke, and/or are experience psychosocial distress.

### Limitations

We recognize several noteworthy limitations to our study methodology. First, ours was an observational study. While randomization would not have been ethical or feasible, propensity scores cannot consider and match for unmeasured confounders. It is possible that unmeasured confounders may have accounted for our results.

Second, BMI as with physical activity was based on self-report and assessed at one point in time, which may have biased our results towards the null. BMI does not take into account central obesity or adiposity, which may be more important prognostic and cost indicators [47]. However, numerous other epidemiologic studies have adopted similar methodology for characterizing body habitus.

Third, medical expenditures were derived from health service utilization encounters, which were captured using administrative data. Only a subset of total expenditures was examined in this study. For example, medical laboratory and other diagnostic imaging tests were not included in our analysis. Moreover, limitations of administrative data include the lack clinical detail and the inability to identify drug-claims for individuals under the age of 65 years (unless an individual’s annual incomes fall below the poverty thresholds). Nonetheless, administrative data for health service utilization is comprehensive, and our cost analysis was conducted from the publicly-funded health care system perspective in accordance with Canadian economic evaluative guidelines [29]. Finally, we did examine data related to the three highest health care-related publicly-funded health-care expenditures in Canada and elsewhere (hospitalizations, physician visits, and medications), which represented a comprehensive estimate of the economic-consequences of obesity on Canada’s publicly-funded health care system.

Finally, our study examined a healthier subset of a representative national population health survey. Ethnic minorities and immigrants were under-represented, and the representative of the population health survey is further limited by the fact that only 30% of the original survey was included in the survey. Moreover, all individuals were free of cardiovascular disease and were younger than 65 years old at inception. Our results might have differed had we examined a population at more at advanced stages of life and disease. However, our intent was to examine a subgroup of the middle-aged population from a time point that preceded the development of disease-related complication, so that we could better disentangle the natural history of obesity from those of other lifestyle behaviours.

## Conclusion

In conclusion, our study demonstrated that the incremental long-term medical expenditures associated with obesity among a middle-aged population are modest as compared with propensity-matched normal weight controls. However, incremental costs markedly rose when obesity (or overweight body mass index) was combined with other concomitant adverse lifestyle behaviours. Such findings reinforce the need for comprehensive health risk stratification and have relevance to the selection, prioritization, and cost-effective targeting of therapeutic lifestyle interventions for policy-makers and system-planners.

## Abbreviations

CAD, Canadian; BMI, Body Mass Index; OHS, Ontario Health Survey; AMI, Acute Myocardial Infarction; NPHS, National Population Health Survey; ODB, Ontario Drug Benefits.

## Competing interests

Dr. Alter is the Scientific Advisor of INTERxVENT Canada, which is a therapeutic lifestyle and disease management company. Dr. Alter has received consultation fees from INTERxVENT Canada in the past 5 years, but no longer does so. Dr. Alter has minimal shares in the organization. INTERxVENT Canada did not finance this article or the article processing charge. Dr. Alter has no other financial competing interests and has no non-financial competing interests to declare. None of the other authors have any financial or non-financial competing interests to declare.

## Authors’ contributions

Dr. A conceived of the study and design, analyzed and drafted the manuscript; Drs. A and S participated in the design of the study and the statistical analysis. AC assisted with statistical analysis. All authors’ interpreted data, read and approved the final manuscript.

## Pre-publication history

The pre-publication history for this paper can be accessed here:

http://www.biomedcentral.com/1472-6963/12/238/prepub

## Supplementary Material

Additional file 1 **Baseline characteristics after propensity matching comparing individuals who were obese (BMI ≥ 30.0) with normal weight individuals (BMI 18.5-24.9), and comparing individuals who were overweight (BMI 25-29.9) with normal weight persons (BMI 18.5-24.9).** (DOC 75 kb) Click here for file

Additional file 2 **Baseline characteristics after propensity matching comparing high-risk with non-high-risk individuals according to the type of risk-factors.** (DOC 206 kb) Click here for file
