# Supplementary material for: Obesity, lifestyle risk-factors, and health service outcomes among healthy middle-aged adults in Canada
Source: BMC Health Serv Res. 2012 Aug 4;12:238. doi: 10.1186/1472-6963-12-238 (PMC3439326; doi:10.1186/1472-6963-12-238)
Supplement: Additional file 1 — Baseline characteristics after propensity matching comparing individuals who were obese (BMI ≥ 30.0) with normal weight individuals (BMI 18.5-24.9), and comparing individuals who were overweight (BMI 25-29.9) with normal weight persons (BMI 18.5-24.9). (DOC 75 kb) [file 1472-6963-12-238-S1.doc]

**Appendix 1:** Baseline characteristics after propensity matching comparing individuals who were obese (BMI ≥ 30.0) with normal

weight individuals (BMI 18.5-24.9), and comparing individuals who were overweight (BMI 25-29.9) with normal weight persons (BMI 18.5-24.9)[[1]](#endnote-2).

|  | **Smoker**  (N=2,966) | **Non-smoker**  (N=2,966) | **Standardized Difference of the mean** |
| --- | --- | --- | --- |
| Male (%) | 1,551 (52.5) | 1,559 (52.8) | 0.01 |
| Mean age (SD) | 38.78 (11.1) | 38.77 (11.8) | 0.00 |
| Caucasian (%) | 2781 (94.2) | 2,830 (95.9) | 0.08 |
| High income (%) | 1040 (35.2) | 1058 (35.8) | 0.01 |
| Intermediate income (%) | 877 (29.7) | 890 (30.1) | 0.01 |
| Low income (%) | 1035 (35.1) | 1004 (34.0) | 0.02 |
| High alcohol consumption (%) | 975 (33.0) | 988 (33.5) | 0.00 |
| Obese (BMI=30+) | 355 (12.0) | 401 (13.6) | 0.05 |
| Overweight (BMI 25.1-29.9) | 1,071 (36.3) | 1,060 (35.9) | 0.01 |
| Normal weight (BMI: 18.5-25.0) | 1,537 (52.1) | 1,480 (50.1) | 0.04 |
| Diabetes (%) | 38 (1.3) | 31 (1.1) | 0.02 |
| Hypertension (%) | 32 (1.1) | 29 (1.0) | 0.01 |
| High depression (%) | 46 (1.6) | 46 (1.6) | 0.00 |
| Current smoking (%) | N/A | N/A | N/A |
| Sedentary | 1,645 (55.5) | 1,660 (56.0) | 0.01 |
| Psychological distress | 1,203 (40.8) | 1,156 (39.2) | 0.03 |
| Prior hospitalizations (%) | 1,046 (35.4) | 992 (33.6) | 0.04 |
|  |  |  |  |
|  | **Distressed**  (N=3,239) | **Non-distressed**  (N=3,329) | **Standardize Differences of the mean** |
| Male (%) | 1,462 (45.1) | 1,453 (44.9) | 0.01 |
| Mean age (SD) | 38.41 (11.28) | 38.21 (11.42) | 0.02 |
| Caucasian (%) | 2,985 (92.2) | 2,996 (92.5) | 0.01 |
| High income (%) | 1,194 (36.9%) | 1,202 (37.1) | 0.01 |
| Intermediate income (%) | 936 (28.9) | 952 (29.4) | 0.01 |
| Low income (%) | 1,109 (34.2) | 1,085 (33.5) | 0.02 |
| Obese (BMI=30+) | 468 (14.4) | 461 (14.2) | 0.01 |
| Overweight (BMI 25.1-29.9) | 1,152 (35.6) | 1,134 (35.0) | 0.01 |
| Normal weight (BMI: 18.5-25.0) | 1,619 (50.0) | 1,644 (50.8) | 0.02 |
| High alcohol consumption (%) | 866 (26.7) | 838 (25.9) | 0.02 |
| Diabetes (%) | 47 (1.5) | 51 (1.6) | 0.01 |
| Hypertension (%) | 42 (1.3) | 32 (1.0) | 0.03 |
| Depression (%) | 43 (1.3) | 43 (1.3) | 0.00 |
| Current smoking (%) | 1,200 (37.0) | 1,189 (36.7) | 0.01 |
| Sedentary | 1,701 (52.5) | 1,809 (54.3) | 0.01 |
| Psychologically distressed | NA | NA |  |
| Prior hospitalizations (%) | 1,207 (37.3) | 1,190 (36.7) | 0.01 |
|  |  |  |  |
|  | **Sedentary**  (N=822) | **Non-sedentary**  (N=822) | **Standardized Difference of the mean** |
| Male (%) | 2,137 (49.8) | 2,147 (50.1) | 0.00 |
| Mean age (SD) | 40.09 (11.4) | 40.08 (12.1) | 0.00 |
| Caucasian (%) | 3,994 (93.1) | 3,998 (93.2) | 0.00 |
| High income (%) | 1,887 (44.0) | 1,889 (44.1) | 0.00 |
| Intermediate income (%) | 1,241 (28.9) | 1,238 (28.9) | 0.00 |
| Low income (%) | 1,148 (27.0) | 1,163 (27.1) | 0.00 |
| High alcohol consumption (%) | 1,164 (27.1) | 1,190 (27.8) | 0.01 |
| Obese (BMI=30+) | 590 (13.8) | 598 (13.9) | 0.01 |
| Overweight (BMI 25.1-29.9) | 1,530 (35.7) | 1,531 (35.7) | 0.00 |
| Normal weight (BMI: 18.5-25.0) | 3,268 (50.6) | 2,159 (50.3) | 0.00 |
| Diabetes (%) | 62 (1.4) | 62 (1.4) | 0.00 |
| Hypertension (%) | 45 (1.0) | 48 (1.1) | 0.01 |
| High depression (%) | 52 (1.2) | 52 (1.2) | 0.00 |
| Current smoking (%) | 1,379 (31.9) | 1,336 (31.2) | 0.02 |
| Sedentary | NA | NA | NA |
| Psychologically distressed | 1,510 (35.2) | 1,518 (35.4) | 0.00 |
| Prior hospitalizations (%) | 1,465 (34.2) | 1,462 (34.1) | 0.00 |
|  |  |  |  |
|  |  |  |  |

1. High alcohol consumption is defined as consumption exceeding the median level for the entire cohort (regardless of subgroup and prior to propensity matching) and consists of consuming ≥ 2 drinks per day. Sedentary lifestyle was defined as a frequency of physical activity that fell below the median for the study sample (i.e,. < 17 days per month of exercising for ≥ 15 minutes per session). High psychological distress is defined as the number of individuals whose distress scores, as measured using the Ontario Health Survey derived distress scale exceeding the median for the entire cohort (a score of >2) regardless of subgroup and prior to propensity matching). [↑](#endnote-ref-2)
