# Supplementary material for: Obesity, lifestyle risk-factors, and health service outcomes among healthy middle-aged adults in Canada
Source: BMC Health Serv Res. 2012 Aug 4;12:238. doi: 10.1186/1472-6963-12-238 (PMC3439326; doi:10.1186/1472-6963-12-238)
Supplement: Additional file 2 — Baseline characteristics after propensity matching comparing high-risk with non-high-risk individuals according to the type of risk-factors. (DOC 206 kb) [file 1472-6963-12-238-S2.doc]

**Appendix 2:** Baseline characteristics after propensity matching comparing high-risk with non-high-risk individuals according to the type of risk-factors.[[1]](#endnote-2)

|  | **Overweight + smoking**  (N=971) | **Normal weight non-smoking controls**  (N=971) | **Standardize Differences of the mean** |
| --- | --- | --- | --- |
| Male (%) | 635 (65.4) | 650 (66.9) | 0.03 |
| Mean age (SD) | 39.9 (11.1) | 40.1 (12.1) | 0.01 |
| Caucasian (%) | 924 (95.2) | 937 (96.5) | 0.07 |
| High income (%) | 376 (38.7) | 390 (40.2) | 0.03 |
| Intermediate income (%) | 291 (30.0) | 285 (29.4) | 0.01 |
| Low income (%) | 304 (31.3) | 296 (30.5) | 0.02 |
| High alcohol consumption (%) | 376 (38.7) | 359 (37.0) | 0.04 |
| Diabetes (%) | 16 (1.6) | 12 (1.2) | 0.03 |
| Hypertension (%) | 11 (1.1) | 8 (0.8) | 0.03 |
| Prior depression (%) | 9 (0.9) | 11 (1.1) | 0.02 |
| Current smoking (%) | N/A | N/A | N/A |
| High physical activity lifestyle | 429 (44.2) | 433 (44.6) | 0.01 |
| Psychological distress | 373 (38.4) | 360 (37.1) | 0.03 |
| Prior hospitalizations (%) | 293 (30.2) | 279 (28.7) | 0.02 |
|  |  |  |  |
|  | **Overweight + Distressed**  (N=1,089) | **Normal weight non-distressed controls**  (N=1,089) | **Standardize Differences of the mean** |
| Male (%) | 634 (58.2) | 658 (60.4) | 0.04 |
| Mean age (SD) | 39.6 (11.3) | 39.5 (12.0) | 0.01 |
| Caucasian (%) | 1,024 (94.0) | 1,034 (94.9) | 0.04 |
| High income (%) | 446 (41.0) | 460 (42.2) | 0.03 |
| Intermediate income (%) | 337 (30.9) | 343 (31.5) | 0.01 |
| Low income (%) | 286 (26.3) | 306 (28.1) | 0.04 |
| High alcohol consumption (%) | 345 (31.7) | 336 (30.9) | 0.02 |
| Diabetes (%) | 20 (1.8) | 18 (1.7) | 0.01 |
| Hypertension (%) | 15 (1.4) | 14 (1.7) | 0.01 |
| Prior depression (%) | 21 (1.9) | 14 (1.3) | 0.05 |
| Current smoking (%) | 385 (35.4) | 360 (33.1) | 0.05 |
| High physical activity lifestyle | 522 (47.9) | 511 (46.9) | 0.02 |
| Psychological distress | N/A | N/A | N/A |
| Prior hospitalizations (%) | 375 (34.4) | 348 (32.0) | 0.05 |
|  |  |  |  |
|  |  |  |  |
|  | **Overweight + sedentary**  (N=1,418) | **Normal weight non-sedentary controls**  (N=1,418) | **Standardize Differences of the mean** |
| Male (%) | 784 (55.3) | 799 (56.3) | 0.02 |
| Mean age (SD) | 40.99 (11.8) | 40.50 (12.1) | 0.04 |
| Caucasian (%) | 1,325 (93.4) | 1,322 (93.2) | 0.01 |
| High income (%) | 629 (44.4) | 635 (44.8) | 0.01 |
| Intermediate income (%) | 409 (28.8) | 398 (28.1) | 0.02 |
| Low income (%) | 380 (26.8) | 385 (27.2) | 0.01 |
| High alcohol consumption (%) | 402 (28.3) | 376 (26.5) | 0.04 |
| Diabetes (%) | 22 (1.6) | 17 (1.2) | 0.03 |
| Hypertension (%) | 10 (0.7) | 9 (0.6) | 0.01 |
| Prior depression (%) | 19 (1.3) | 17 (1.2) | 0.03 |
| Current smoking (%) | 451 (31.8) | 456 (32.2) | 0.01 |
| High physical activity lifestyle | N/A | N/A | N/A |
| Psychological distress | 476 (33.6) | 495 (34.9) | 0.03 |
| Prior hospitalizations (%) | 445 (31.4) | 438 (30.9) | 0.01 |
|  |  |  |  |
|  | **Overweight + smoking + distress**  (N=380) | **Normal weight, non-smoking, non-distressed controls**  (N=380) | **Standardize Differences of the mean** |
| Male (%) | 236 (62.1) | 235 (61.8) | 0.01 |
| Mean age (SD) | 38.6 (10.77) | 38.9 (11.65) | 0.03 |
| Caucasian (%) | 363 (95.5) | 365 (96.1) | 0.03 |
| High income (%) | 120 (31.6) | 117 (30.8) | 0.02 |
| Intermediate income (%) | 123 (32.4) | 121 (31.8) | 0.01 |
| Low income (%) | 137 (36.1) | 142 (37.4) | 0.03 |
| High alcohol consumption (%) | 145 (38.2) | 149 (39.2) | 0.02 |
| Diabetes (%) | 8 (2.1) | 8 (2.1) | 0.00 |
| Hypertension (%) | 6 (1.6) | 3 (0.8) | 0.07 |
| Prior depression (%) | 8 (2.1) | 6 (1.6) | 0.04 |
| Current smoking (%) | N/A | N/A | N/A |
| High physical activity lifestyle | 165 (43.4) | 165 (43.4) | 0.00 |
| Psychologically distressed | N/A | N/A | N/A |
| Prior hospitalizations (%) | 122 (32.1) | 110 (28.9) | 0.07 |
|  |  |  |  |
|  | **Overweight + smoking + sedentary**  (N=535) | **Normal weight non-smoking, non-sedentary controls**  (N=535) | **Standardize Differences of the mean** |
| Male (%) | 347 (64.9) | 363 (67.9) | 0.06 |
| Mean age (SD) | 39.9 (11.1) | 39.99 (12.7) | 0.01 |
| Caucasian (%) | 503 (94.0) | 506 (94.6) | 0.02 |
| High income (%) | 206 (38.5) | 213 (39.8) | 0.03 |
| Intermediate income (%) | 169 (31.6) | 157 (29.3) | 0.05 |
| Low income (%) | 165 (30.8) | 160 (29.9) | 0.02 |
| High alcohol consumption (%) | 206 (38.5) | 191 (35.7) | 0.06 |
| Diabetes (%) | 10 (1.9) | 7 (1.3) | 0.04 |
| Hypertension (%) | 7 (1.3) | 2 (0.04) | 0.1 |
| Prior depression (%) | 7 (1.3) | 5 (0.9) | 0.04 |
| Current smoking (%) | N/A | N/A | N/A |
| High physical activity lifestyle | N/A | N/A | N/A |
| Psychologically distressed | 208 (38.9) | 187 (35.0) | 0.08 |
| Prior hospitalizations (%) | 150 (28.0) | 142 (26.5) | 0.03 |
|  | **Overweight + sedentary + distress**  (N=566) | **Normal weight non-sedentary, non-distressed controls**  (N=566) | **Standardize Differences of the mean** |
| Male (%) | 311 (54.9) | 332 (58.7) | 0.07 |
| Mean age (SD) | 39.86 (11.1) | 39.68 (12.1) | 0.02 |
| Caucasian (%) | 529 (93.5) | 529 (93.5) | 0.00 |
| High income (%) | 220 (38.9) | 227 (40.1) | 0.03 |
| Intermediate income (%) | 181 (32.0) | 175 (30.9) | 0.02 |
| Low income (%) | 165 (29.2) | 164 (29.0) | 0.00 |
| High alcohol consumption (%) | 164 (29.0) | 163 (28.8) | 0.00 |
| Diabetes (%) | 9 (1.6) | 6 (1.1) | 0.05 |
| Hypertension (%) | 9 (1.6) | 6 (1.1) | 0.05 |
| Prior depression (%) | 11 (1.9) | 7 (1.2) | 0.06 |
| Current smoking (%) | 218 (38.5) | 212 (37.5) | 0.02 |
| High physical activity lifestyle | N/A | N/A | N/A |
| Psychologically distressed | N/A | N/A | N/A |
| Prior hospitalizations (%) | 194 (34.3) | 177 (31.3) | 0.06 |
|  |  |  |  |
|  | **Obese + smoking**  (N=381) | **Normal weight non-smoking controls**    (N=381) | **Standardize Differences of the mean** |
| Male (%) | 202 (53) | 212 (55.6) | 0.05 |
| Mean age (SD) | 40.2 (10.6) | 39.7 (12.2) | 0.04 |
| Caucasian (%) | 358 (94) | 367 (96.3) | 0.11 |
| High income (%) | 127 (33.3) | 135 (35.4) | 0.04 |
| Intermediate income (%) | 108 (28.3) | 107 (28.1) | 0.01 |
| Low income (%) | 146 (38.3) | 139 (36.5) | 0.04 |
| High alcohol consumption (%) | 104 (27.3) | 95 (24.9) | 0.05 |
| Diabetes (%) | 6 (1.6) | 5 (1.3) | 0.02 |
| Hypertension (%) | 7 (1.8) | 7 (1.8) | 0.00 |
| Prior depression (%) | 5 (1.3) | 4 (1.0) | 0.02 |
| Current smoking (%) | N/A | N/A | N/A |
| High physical activity lifestyle | 152 (39.9) | 150 (39.4) | 0.01 |
| Psychological distress | 170 (44.6) | 168 (44.1) | 0.01 |
| Prior hospitalizations (%) | 136 (35.7) | 122 (32.0) | 0.08 |
|  |  |  |  |
|  | **Obese + distressed**  (N=508) | **Normal weight non-distressed controls** (N=508) | **Standardize Differences of the mean** |
| Male (%) | 207 (40.7) | 205 (40.4) | 0.01 |
| Mean age (SD) | 41.1 (11.23) | 41.01 (11.83) | 0.01 |
| Caucasian (%) | 477 (93.9) | 474 (93.3) | 0.02 |
| High income (%) | 148 (29.1) | 147 (28.9) | 0.00 |
| Intermediate income (%) | 148 (29.1) | 160 (31.5) | 0.05 |
| Low income (%) | 212 (41.7) | 201 (39.6) | 0.04 |
| High alcohol consumption (%) | 108 (21.3) | 87 (17.1) | 0.11 |
| Diabetes (%) | 11 (2.2) | 8 (1.6) | 0.04 |
| Hypertension (%) | 9 (1.8) | 9 (1.8) | 0.00 |
| Prior depression (%) | 13 (2.6) | 15 (3.0) | 0.02 |
| Current smoking (%) | 172 (33.9) | 171(33.7) | 0.00 |
| High physical activity lifestyle | 203 (40.0) | 189 (37.2) | 0.06 |
| Psychological distress | N/A | N/A | N/A |
| Prior hospitalizations (%) | 200 (39.4) | 191 (37.6) | 0.04 |
|  |  |  |  |
|  |  |  |  |
|  | **Obese + sedentary**  (N=765) | **Normal weight non-sedentary controls**  (N=765) | **Standardize Differences of the mean** |
| Male (%) | 410 (53.6) | 423 (55.3) | 0.03 |
| Mean age (SD) | 42.9 (11.1) | 42.8 (11.9) | 0.01 |
| Caucasian (%) | 733 (95.8) | 736 (96.2) | 0.02 |
| High income (%) | 317 (41.4) | 318 (41.6) | 0.00 |
| Intermediate income (%) | 216 (28.2) | 219 (28.6) | 0.01 |
| Low income (%) | 232 (30.3) | 228 (29.8) | 0.01 |
| High alcohol consumption (%) | 185 (24.2) | 170 (22.2) | 0.05 |
| Diabetes (%) | 17 (2.2) | 10 (1.3) | 0.07 |
| Hypertension (%) | 14 (1.8) | 9 (1.2) | 0.02 |
| Prior depression (%) | 11 (1.4) | 11 (1.4) | 0.00 |
| Current smoking (%) | 227 (29.7) | 231 (30.2) | 0.01 |
| High physical activity lifestyle | N/A | N/A | N/A |
| Psychological distress | 306 (40.0) | 310 (40.5) | 0.01 |
| Prior hospitalizations (%) | 278 (36.3) | 259 (33.9) | 0.01 |
|  |  |  |  |
|  | **Obese + smoking + distress**  (N=167) | **Normal weight, non-smoking, non-distressed controls**  (N=167) | **Standardize Differences of the mean** |
| Male (%) | 66 (39.5) | 63 (37.7) | 0.04 |
| Mean age (SD) | 39.87 (10.88) | 39.68 (11.09) | 0.02 |
| Caucasian (%) | 151 (90.4) | 152 (91.0) | 0.02 |
| High income (%) | 40 (24.0) | 37 (22.2) | 0.04 |
| Intermediate income (%) | 44 (26.3) | 47 (28.1) | 0.04 |
| Low income (%) | 83 (49.7) | 83 (49.7) | 0.00 |
| High alcohol consumption (%) | 43 (25.7) | 42 (25.1) | 0.01 |
| Diabetes (%) | 2 (1.2) | 6 (3.6) | 0.16 |
| Hypertension (%) | 4 (2.4) | 2 (1.2) | 0.09 |
| Prior depression (%) | 4 (2.4) | 3 (1.8) | 0.04 |
| Current smoking (%) | N/A | N/A | N/A |
| High physical activity lifestyle | 71 (42.5) | 79 (47.3) | 0.10 |
| Psychologically distressed | N/A | N/A | N/A |
| Prior hospitalizations (%) | 66 (39.5) | 71 (42.5) | 0.06 |
|  |  |  |  |
|  | **Obese + smoking + sedentary**  (N=229) | **Normal weight, non-smoking, non-sedentary controls** (N=229) | **Standardize Differences of the mean** |
| Male (%) | 121 (52.8) | 125 (54.6) | 0.03 |
| Mean age (SD) | 40.99 (10.63) | 41.10 (11.25) | 0.01 |
| Caucasian (%) | 216 (94.3) | 221 (96.5) | 0.10 |
| High income (%) | 79 (34.5) | 88 (38.4) | 0.08 |
| Intermediate income (%) | 63 (27.5) | 57 (24.9) | 0.06 |
| Low income (%) | 87 (38.0) | 84 (36.7) | 0.03 |
| High alcohol consumption (%) | 61 (26.6) | 63 (27.5) | 0.02 |
| Diabetes (%) | 3 (1.3) | 4 (1.7) | 0.04 |
| Hypertension (%) | 6 (2.6) | 5 (2.2) | 0.03 |
| Prior depression (%) | 3 (1.3) | 6 (2.6) | 0.09 |
| Current smoking (%) | N/A | N/A | N/A |
| High physical activity lifestyle | N/A | N/A | N/A |
| Psychologically distressed | 98 (42.8) | 97 (42.4) | 0.01 |
| Prior hospitalizations (%) |  |  |  |
|  |  |  |  |
|  | **Obese + sedentary + distress**  (N=302) | **Normal weight, non-sedentary, non-distressed controls**  (N=302) | **Standardize Differences of the mean** |
| Male (%) | 123 (40.7) | 135 (44.7) | 0.08 |
| Mean age (SD) | 41.87 (11.27) | 42.34 (12.4) | 0.04 |
| Caucasian (%) | 287 (95.0) | 289 (95.7) | 0.03 |
| High income (%) | 87 (28.8) | 95 (31.5) | 0.06 |
| Intermediate income (%) | 90 (28.8) | 89 (29.5) | 0.01 |
| Low income (%) | 125 (41.4) | 118 (39.1) | 0.05 |
| High alcohol consumption (%) | 67 (22.2) | 60 (19.9) | 0.06 |
| Diabetes (%) | 7 (2.3) | 4 (1.3) | 0.07 |
| Hypertension (%) | 4 (1.3) | 6 (2.0) | 0.05 |
| Prior depression (%) | 6 (2.0) | 9 (3.0) | 0.06 |
| Current smoking (%) | 95 (31.5) | 92 (30.5) | 0.02 |
| High physical activity lifestyle | N/A | N/A | N/A |
| Psychologically distressed | N/A | N/A | N/A |
| Prior hospitalizations (%) | 120 (39.7) | 106 (35.1) | 0.10 |

1. High alcohol consumption is defined as consumption exceeding the median level for the entire cohort (regardless of subgroup and prior to propensity matching) and consists of consuming ≥ 2 drinks per day. Sedentary lifestyle was defined as a frequency of physical activity that fell below the median for the study sample (i.e,. < 17 days per month of exercising for ≥ 15 minutes per session). High psychological distress is defined as the number of individuals whose distress scores, as measured using the Ontario Health Survey derived distress scale exceeding the median for the entire cohort (a score of >2) regardless of subgroup and prior to propensity matching). [↑](#endnote-ref-2)
